# *β*-Carotene Attenuates Apoptosis and Autophagy via PI3K/AKT/mTOR Signaling Pathway in Necrotizing Enterocolitis Model Cells IEC-6

**DOI:** 10.1155/2022/2502263

**Published:** 2022-06-17

**Authors:** Guang Xu, Tidong Ma, Chonggao Zhou, Fan Zhao, Kun Peng, Bixiang Li

**Affiliations:** Department of Neonatal Surgery, Hunan Children's Hospital, Changsha 410007, China

## Abstract

**Background:**

Necrotizing enterocolitis (NEC) is a devastating disease affecting the gastrointestinal tract in the newborn period. In recent years, the role of apoptosis and autophagy in intestinal mucosal barrier dysfunction has come into prominence in research regarding the pathogenesis of NEC. *β*-Carotene is a well-known vitamin A precursor, and its content in breast milk is relatively high, especially in the colostrum. In the present study, we investigated the protective effect of *β*-carotene on necrotizing enterocolitis model cells IEC-6 induced by lipopolysaccharide (LPS).

**Methods:**

CCK-8 assay was performed to evaluate cell viability. The Annexin V-FITC/PI method was used to detect apoptosis. Western blotting was utilized to measure the expression levels of proteins. Immunofluorescence analysis was used to assess the autophagy of IEC-6 cells.

**Results:**

Our findings indicated that *β*-carotene inhibited the apoptosis of IEC-6 cells by downregulating cleaved caspase-3 levels and Bax levels and upregulating Bcl-2 levels, reducing cell autophagy via downregulating LC3II/I ratio and upregulating p62 levels. In addition, the expression of p-PI3K, p-AKT, and p-mTOR was upregulated after *β*-carotene treatment. Interestingly, these changes induced by *β*-carotene were partially reversed by rapamycin and voxtalisib.

**Conclusion:**

In conclusion, our findings indicated that *β*-carotene can attenuate apoptosis and autophagy of IEC-6 cells induced by LPS via activating the PI3K/AKT/mTOR signaling pathway. Therefore, *β*-carotene may be a promising drug used in the clinical treatment of NEC.

## 1. Background

Necrotizing enterocolitis (NEC) is the most common gastrointestinal emergency occurring in the neonatal period. In the general population, the incidence of NEC is about 1.1 per 1000 live births, but it affects up to 5–7% of NEC among very low birth weight infants [1, 2]. Despite progress in neonatal intensive care and a greater understanding of the pathophysiology of this disease, the total mortality rate of NEC remains between 25% and 40% [2]. Furthermore, 30–50% of NEC cases require surgical treatment, with a mortality rate of 40–50% [3]. In addition, due to intestinal diseases and other complications of recovered infants, NEC patients may still require long-term hospitalization for cholestasis, short bowel syndrome, complete intestinal failure, and impaired neurodevelopment, which will further affect their long-term survival, growth, and development [4].

The role of apoptosis and autophagy in intestinal mucosal barrier dysfunction has become a focal point in NEC pathogenesis [5–7]. Epithelial cell apoptosis is the most common feature of NEC patients [8]. Experimental studies showed that alterations of apoptosis in intestinal epithelial cells tend to affect NEC development [9]. Bcl-2 and epidermal growth factor can reduce the apoptosis of intestinal epithelial cells and protect the intestinal epithelium from NEC damage [10]. Increasing studies have shown that the dysregulation of autophagy is a susceptible factor for many intestinal diseases. Inflammatory reactions are regulated by autophagy, and autophagy can effectively alleviate inflammatory reactions [11]. Previous research has shown that autophagy is upregulated in the rat NEC model and in patients suffering from NEC [12, 13].

*β*-Carotene is the precursor of vitamin A and widely exists in green plants, fruits, and vegetables [14]. *β*-Carotene is widely recognized as a powerful scavenger of ROS, which has many biological characteristics such as anticancer, antioxidation, anti-inflammation, and immunomodulation [15]. *β*-Carotene can inhibit the proliferation of cancer cells such as gastric cancer, colon cancer, breast cancer, and esophageal cancer and promote the apoptosis of tumor cells [16–21]. A recent study indicates that *β*-carotene exerts strong anti-inflammatory activity by inhibiting the expression of Tnfa, Nos 2, and Cox2 genes and upregulating the expression of Hmox 1 gene [22]. In addition, *β*-carotene is one of the most important nutrients in breast milk, and its content is relatively high in the colostrum [23, 24]. Breast-feeding has a protective effect on newborns, which can effectively reduce the incidence of NEC, and the role played by its components has always been the focus of NEC research [25].

However, the effect of *β*-carotene on apoptosis and autophagy of intestinal epithelial cells in NEC patients has not been reported. Therefore, this study aimed to explore the effect of *β*-carotene on lipopolysaccharide (LPS)-induced apoptosis and autophagy of NEC model cells IEC-6 and to explore the potential mechanism to provide an experimental basis for clinical prevention and treatment of NEC.

## 2. Methods

### 2.1. Cell Lines and Cell Culture

The IEC-6 cells used in this experiment were purchased from the Cell Bank of the Chinese Academy of Sciences (Shanghai, China). IEC-6 cells were grown in an incubator in Dulbecco's modified Eagle's medium (DMEM, Gibco BRL, Gaithersburg, MD, USA) complemented with 10% fetal bovine serum (FBS, Gibco BRL), streptomycin (100 *μ*g/mL), penicillin (100 U/mL), and recombinant human insulin (0.1 U/mL) at 37°C in 5% CO_2_.

### 2.2. Chemicals and Reagents

*β*-Carotene was purchased from MedChem Express (MCE Co., Ltd., Shanghai, China). LPS and rapamycin (RAPA) were obtained from Sigma Company (St. Louis, MO, USA). Culture medium (DMEM) was furnished by Gibco BRL (Carlsbad, CA, USA). Fetal bovine serum, pancreatic digestive juice, DPBS, and CCK-8 kits were supplied by Beyotime Institute of Biotechnology (Haimen, China). The FITC Annexin V apoptosis detection kit I was obtained from BD PharMingen (San Diego, CA). The stubRFP-sensGFP-LC3 adenovirus was provided by JikaiGene (Shanghai, China). Primary antibodies against cleaved caspase-3, Bcl-2, Bax, p62, LC3II/I, p-PI3K, PI3K, p-AKT, AKT, p-mTOR, mTOR, and *β*-actin were purchased from Wuhan Sanying Biotechnology (Wuhan, China).

### 2.3. Cell Counting Kit-8 (CCK-8) Assay

Cell proliferation was evaluated using the Cell Counting Kit-8 (CCK-8) assay. IEC-6 cells (1 × 10^4^ cells/well) in a logarithmic growth phase were seeded into 96-well plates at a density of 5000 cells/well, and five auxiliary wells were set for each group. The cells adhered to the wall after 24 h of incubation. The next day, the original medium with media containing different drugs, LPS (100 *μ*g/ml), *β*-carotene (0–100 *μ*M), rapamycin (10 *μ*M), and voxtalisib (5 *μ*M) was replaced, and the cells were incubated for 24 h at 37°C under 5% CO_2_. Thereafter, 100 *μ*L of CCK-8 reagent was added to each well, and the plates were incubated for 4 h. Finally, cell viability was determined by measuring the absorbance of the samples at 450 nm with a microplate reader (BioRad, Hercules, CA, USA).

### 2.4. Annexin V-Fluorescein Isothiocyanate (FITC)/Propidium Iodide (PI) Dual Staining Assay

Following 24 h of treatment with different drugs, IEC-6 cells were washed twice with ice-cold phosphate-buffered saline (PBS), resuspended in Annexin V binding buffer at 1 × 10^5^ cells/ml, and 100 *μ*l of the solution was drawn out and transferred to 6-well culture plates (5 ml). Then, incubation with fluorescein isothiocyanate (FITC)-conjugated Annexin V antibody and propidium iodide occurred for 15 min at room temperature in the dark. Data analysis was performed using FlowJo (Tree Star, Ashland, OR, USA). FITC and PI fluorescence were analyzed on a FACSort flow cytometer (BD Biosciences, San Diego, CA, USA) with the CellQuest Pro software (FACSstation 6.0 BD Biosciences, Franklin Lakes, NJ, USA).

### 2.5. Protein Extraction and Western Blot Analysis

The treated IEC-6 cells were lysed for 30 min in an ice-cold RIPA solution containing 1% PMSF and 1% phosphorylated inhibitors. The protein content was measured by the BCA Protein Assay (Thermo Scientific, Fremont, USA). The total protein sample was electrophoresed through 10% SDS-PAGE and transferred to PVDF membranes. The membranes were blocked with 5% nonfat milk in TBST buffer at room temperature for 1 h and subsequently incubated with primary antibodies at 4°C overnight. After washing three times with TBST buffer, the membranes were incubated with secondary antibodies (1 : 1,000; G130321; Hangzhou HuaAn Biotechnology Co., Ltd., Hangzhou, China) for 2 h at room temperature. *β*-Actin was selected as the internal control. An imaging system (BioRad, USA) was used to scan the obtained blots. The intensity of protein bands was semiquantified using the image analysis software (Image Processing and Analysis in Java; NIH, Bethesda, MD, USA).

### 2.6. Measurements of Autophagy Puncta

The IEC-6 cells were seeded at a density of 1 × 10^5^ cells in 6-well plates and cultured in a 5% CO_2_ cell incubator at 37°C, and after overnight culture, cells were transduced with the stubRFP-sensGFP-LC3 adenovirus in serum-free medium at a multiplicity of infection (MOI) of 100. Subsequent to infection for 24 h, the supernatant of the culture medium was discarded, and each group was replaced with a fresh culture medium. After changing the solution for 24 hours, the control group was replaced with a normal culture medium, and the other groups were treated with drugs and then photographed under a fluorescence microscope (Nikon America Inc., Melville, NY).

### 2.7. Statistical Analyses

The SPSS 23.0 software (SPSS Inc., Chicago, IL, USA) was used for analysis. Data were expressed as mean ± SD. A one-way analysis of variance (ANOVA) or *t*-test was performed to analyze statistical significance.  ^*∗*^*P* < 0.05 was considered statistically significant.

## 3. Results

### 3.1. Effect of *β*-Carotene on the Viability of IEC-6 Cells

**Figure 1:**
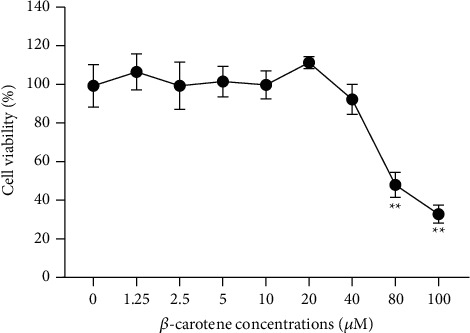
The CCK-8 assay was used to measure the viability of IEC-6 cells. IEC-6 cells were treated with *β*-carotene at the indicated doses (0, 1.25, 2.5, 5, 10, 20, 40, 80, and 100 *μ*M) for 24 h.  ^*∗*^ ^*∗*^*P* < 0.01 vs. the 0 *μ*M group.

The cytotoxic effect of *β*-carotene on IEC-6 cells was first evaluated by the CCK-8 assay. *β*-Carotene up to 40 *μ*M did not obviously affect cell viability. However, obvious toxicity and growth inhibition of IEC-6 cells began to appear when the concentration reached more than 40 *μ*M, and the cell viability was gradually reduced in a dose-dependent manner (Figure 1). These facts inspired us to further investigate the inhibitory effects of *β*-carotene on the apoptosis and autophagy of IEC-6 cells.

### 3.2. Effects of *β*-Carotene at Different Concentrations on Apoptosis and Autophagy in LPS-Treated IEC-6 Cells

**Figure 2:**
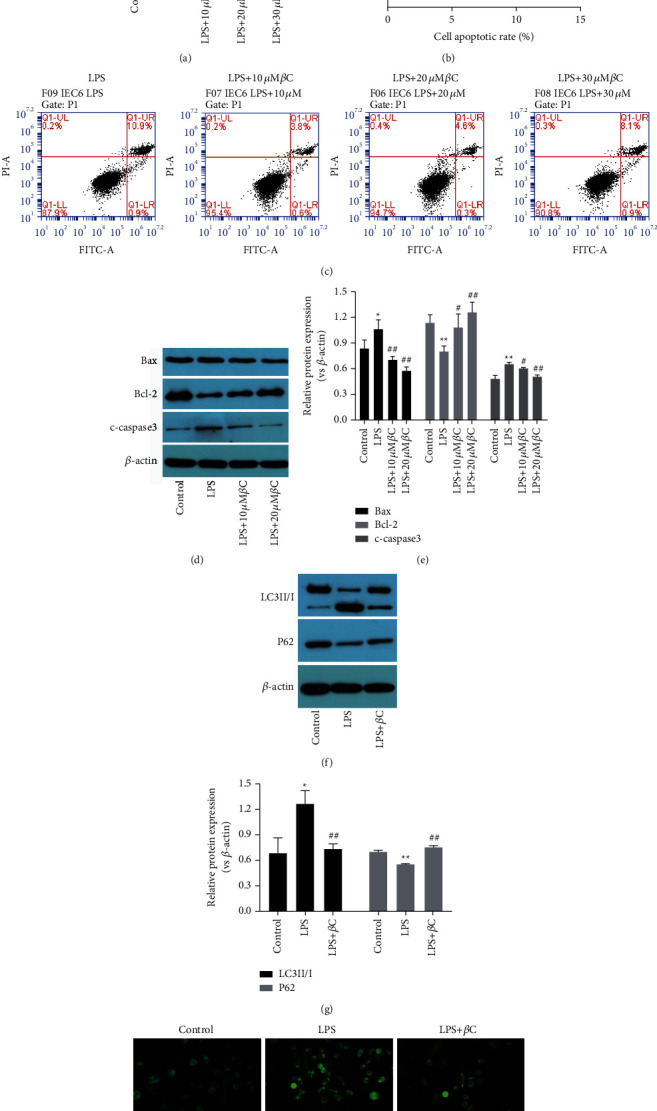
*β*-Carotene inhibits LPS-induced apoptosis and autophagy in IEC-6 cells. Cells were treated with LPS (100 *μ*M) for 3 h prior to exposure to *β*-carotene (10, 20, and 30 *μ*M) for 24 h. (a) Cell viability measured by the CCK-8 assay.  ^*∗*^ ^*∗*^*P* < 0.01 vs. the control group. ^##^*P* < 0.01 vs. the LPS group. (b), (c) Apoptosis was quantified by flow cytometry.  ^*∗*^ ^*∗*^*P* < 0.01 vs. the LPS group. ^##^*P* < 0.01 vs. the LPS + 20 *μ*M *β*C group. (d)–(g) The expressions of Bax, Bcl-2, cleaved caspase-3, LC3II/I, and p62 detected by Western blot.  ^*∗*^*P* < 0.05,  ^*∗*^ ^*∗*^*P* < 0.01 vs. the LPS group. ^#^*P* < 0.05, ^##^*P* < 0.01 vs. the LPS group. (h) Representative images of intracellular GFP-LC3 puncta in IEC-6 cells under different conditions. The images were made at × 100 magnification. Each experiment is carried out in triplicate.

The cell viability of LPS-treated IEC-6 cells was remarkably decreased, compared with the normal cultured cells. However, the cell viability of LPS-treated cells was significantly restored following *β*-carotene (10–30 *μ*M) treatment, as shown in Figure 2(a). This result demonstrated that, to a certain extent, *β*-carotene improved the survival of IEC-6 cells under the pressure caused by LPS. To gain insight into the alleviation effect of *β*-carotene on LPS-induced apoptosis in IEC-6 cells, the Annexin V-FITC/PI assay was applied to measure cell apoptosis. As shown in Figures 2(b) and 2(c), compared with the LPS group, the apoptotic rate was markedly decreased after *β*-carotene treatment at the concentrations of 10, 20, and 30 *μ*M for 24 h. However, the apoptosis rate of the 30 *μ*M *β*-carotene group was higher than that of the 20 *μ*M group. Therefore, we chose 10 and 20 *μ*M *β*-carotene in subsequent experiments. Next, apoptosis-related proteins were detected using a Western blot. The proapoptotic proteins Bax and cleaved caspase-3 were significantly decreased, and the apoptosis inhibitory protein Bcl-2 was increased in the *β*-carotene treatment group when compared with the LPS group (Figures 2(d) and 2(e)). These results again suggested that *β*-carotene could reduce the IEC-6 apoptosis caused by LPS. To explore whether the protective effect of *β*-carotene on cell survival was related to autophagy, autophagy markers were detected using Western blot also. The results showed that the LC3II/I ratio was enhanced, and the autophagy substrate p62 decreased obviously when the cell was stimulated by LPS, which demonstrated that LPS stimulation increased autophagy in IEC-6 cells. However, when LPS-stimulated IEC-6 cells were treated with *β*-carotene, the originally increased LC3II/I proportion and reduced p62 expression were both restored to a certain extent (Figures 2(f) and 2(g)). Moreover, we used a tandem stubRFP-sensGFP-LC3 adenovirus to detect the autophagy puncta following. IEC-6 cells in the LPS group presented an increased accumulation of green autophagy puncta compared with the control group, while the increased number of green autophagy puncta decreased after intervention with 10 *μ*M *β*-carotene (Figure 2(h)). Overall, these results revealed that *β*-carotene suppressed apoptosis and autophagy in LPS-induced IEC-6 cells.

### 3.3. The Protective Effect of *β*-Carotene Is Associated with Inhibited Autophagy in LPS-Treated IEC-6 Cells

**Figure 3:**
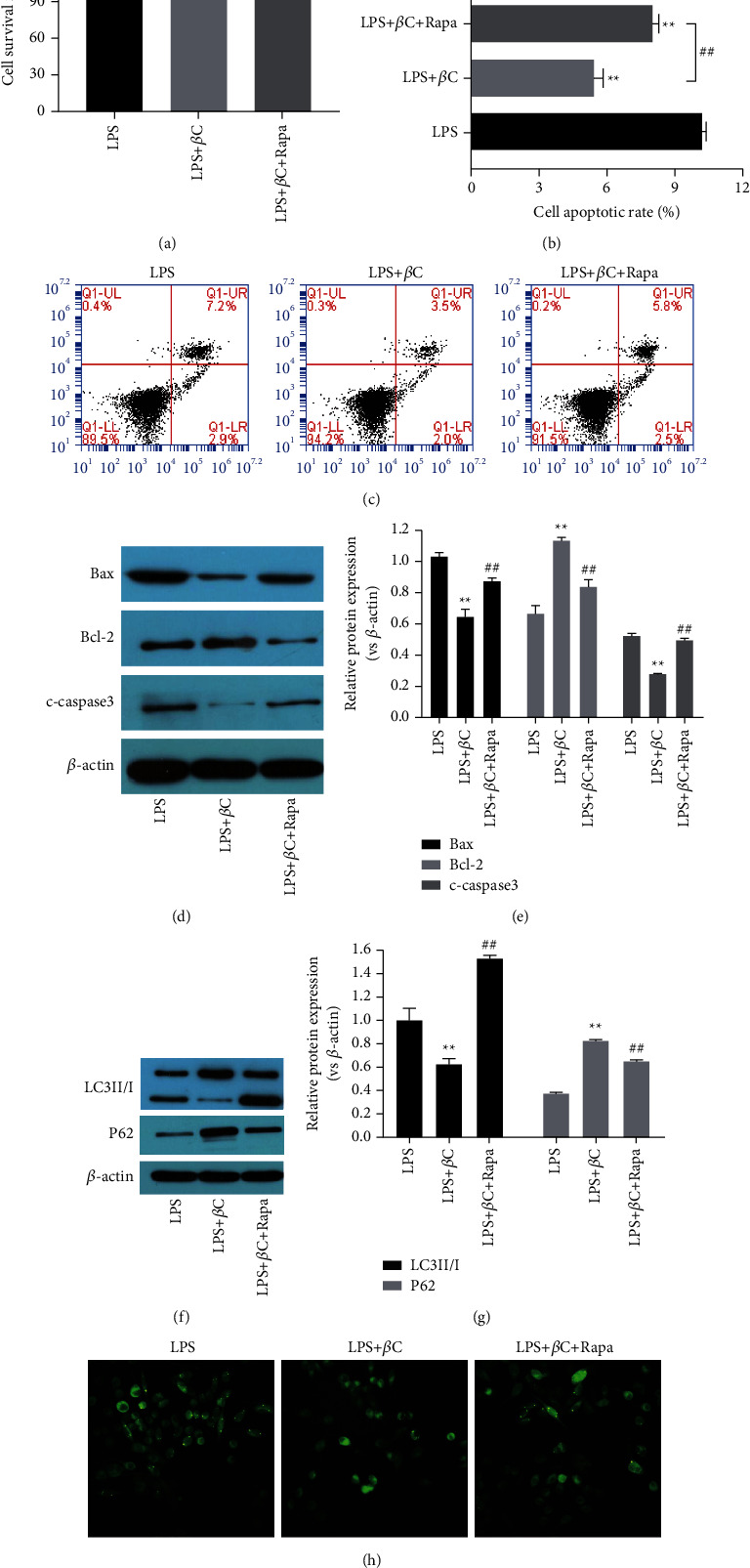
Protective effect of *β*-carotene is related to inhibited autophagy in LPS-treated IEC-6 cells. Cells were exposed to LPS (100 *μ*M) for 3 h followed by incubation with *β*-carotene (10 *μ*M) with or without RAPA (10 *μ*M) for 24 h. (a) Cell viability measured by the CCK-8 assay. (b), (c) Apoptosis quantified by flow cytometry. (d)–(g) The protein expressions of Bax, Bcl-2, cleaved caspase-3, LC3II/I, and p62 detected by Western blot. (h) The formation of GFP-LC3 puncta detected by immunofluorescence. The images were made at × 100 magnification.  ^*∗*^ ^*∗*^*P* < 0.01 vs. the LPS group. ^##^*P* < 0.01 vs. the LPS + *β*C group.

To determine whether the protection of *β*-carotene in LPS-stimulated IEC-6 cells is mediated through autophagy regulation, the cells were exposed to 10 *μ*M rapamycin (an autophagy agonist) when treated with *β*-carotene. Notably, the CCK-8 results showed that the ability of *β*-carotene to promote IEC-6 cell viability was markedly attenuated by rapamycin (Figure 3(a)). IEC-6 cell apoptosis was downregulated in the LPS + *β*C group, while it was upregulated in the LPS + *β*C + RAPA group compared with the LPS group (Figures 3(b) and 3(c)). Moreover, Western blot results revealed that the apoptosis-related proteins of Bax, cleaved caspase-3, were significantly increased, while Bcl-2 was decreased after treatment with rapamycin in comparison with the LPS + *β*C group (Figures 3(d) and 3(e)), which suggested that rapamycin could reverse the inhibition of *β*-carotene on IEC-6 cells apoptosis to a certain extent. Subsequently, cell autophagy was assessed. IEC-6 cells in the LPS + *β*C group presented a relatively lower LC3II/I ratio, higher p62 expression, and decreased accumulation of green autophagy puncta compared with the LPS group. Interestingly, these changes induced by *β*-carotene were partially reversed after intervention with rapamycin (Figures 3(f)–3(h)). Taken together, these results demonstrated that *β*-carotene could relieve apoptosis in necrotizing enterocolitis model cells IEC-6 by inhibiting the activation of proapoptotic autophagy.

### 3.4. *β*-Carotene Attenuates LPS-Induced Apoptosis and Autophagy via Activation of the PI3K/AKT/mTOR Signaling Pathway in IEC-6 Cells

**Figure 4:**
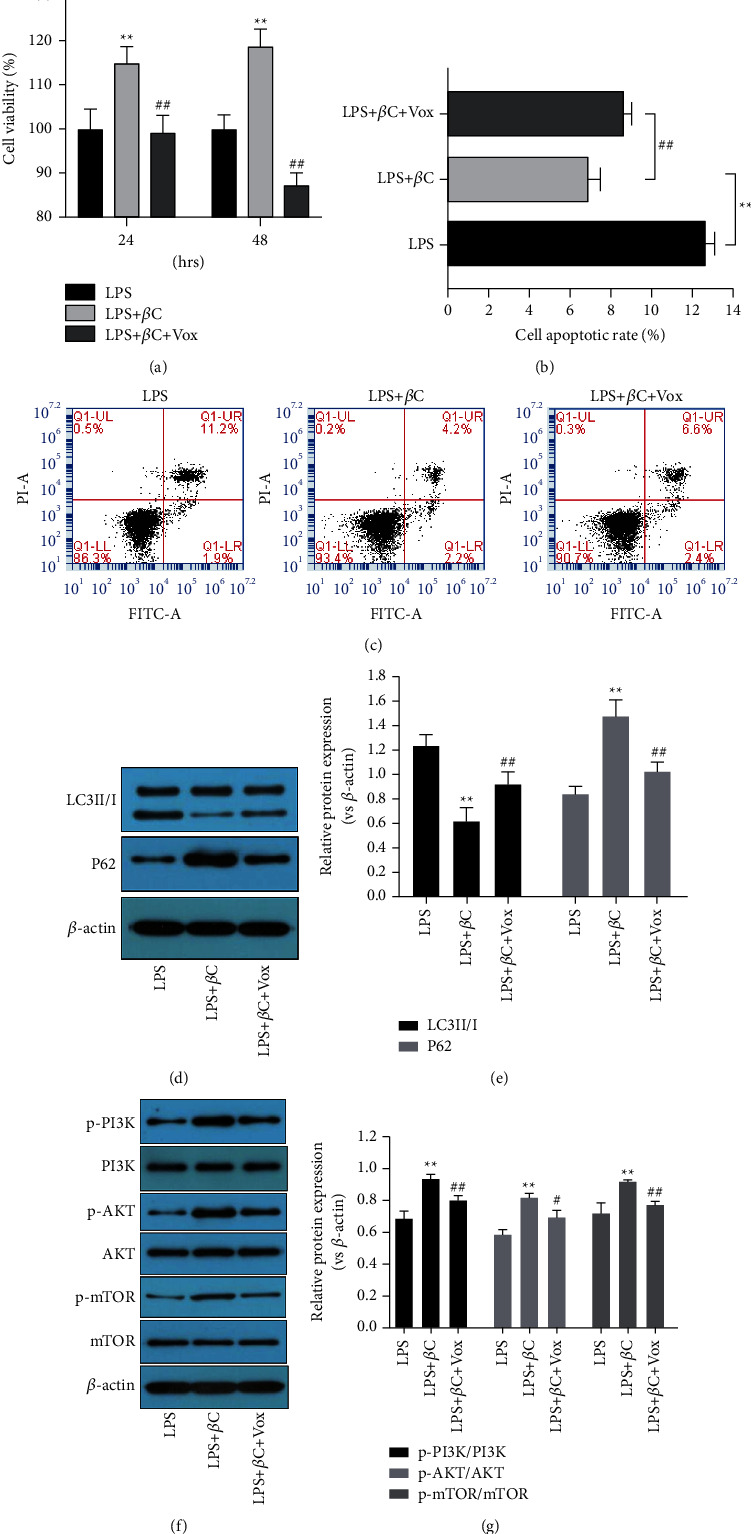
*β*-Carotene induces LPS-induced apoptosis and autophagy via activation of the PI3K/AKT/mTOR signaling pathway in IEC-6 cells. Cells were pretreated with LPS (100 *μ*M) for 3 h following incubation with *β*-carotene (10 *μ*M) with or without voxtalisib (5 *μ*M) for 24 h. (a) Cell viability measured by the CCK-8 assay. (b), (c) Apoptosis quantified by flow cytometry. (d), (e) The protein expressions of LC3II/I and p62 observed by Western blot. (f), (g) Western blot analysis for the expression of p-PI3K, PI3K, p-AKT, AKT, mTOR, and p-mTOR.  ^*∗*^ ^*∗*^*P* < 0.01 vs. the LPS group. ^#^*P* < 0.05, ^##^*P* < 0.01 vs. the LPS + *β*C group.

To verify whether *β*-carotene suppresses LPS-induced apoptosis and autophagy via the PI3K/AKT/mTOR pathway, we exposed the IEC-6 cells to *β*-carotene with or without 5 *μ*M voxtalisib (a PI3K inhibitor) after treatment with LPS. As shown in Figure 4(a), the CCK-8 results showed that the ability of *β*-carotene to promote IEC-6 cell growth was markedly decreased by treatment with voxtalisib for 24 h or 48 h and in a time-dependent manner. The downregulation of apoptosis induced by *β*-carotene in IEC-6 cells was partially reversed by inhibiting the PI3K/AKT/mTOR pathway with voxtalisib (Figures 4(b) and 4(c)). *β*-Carotene and voxtalisib cotreated caused a significant reduction in p62 expression but upregulated the protein expression of LC3II/I compared with the LPS + *β*C group (Figures 4(d) and 4(e)). These results indicate that *β*-carotene could alleviate LPS-induced apoptosis and autophagy in IEC-6 cells, and this effect can be partially reversed by voxtalisib. As shown in Figures 4(f) and 4(g), treatment with *β*-carotene notably increased the expression of p-PI3K, p-AKT, and p-mTOR. Interestingly, *β*-carotene and voxtalisib cotreated significantly downregulated the expression of p-PI3K, p-AKT, and p-mTOR. Therefore, these results proved that *β*-carotene could attenuate apoptosis and autophagy of IEC-6 cells through activating the PI3K/AKT/mTOR signaling pathway.

## 4. Discussion

The exact pathogenesis of NEC remains largely unclear, but the main risk factors, such as intestinal hypoxia, formula feeding, abnormal bacterial colonization, and prematurity, are acknowledged to contribute to the occurrence and development of NEC [26]. NEC can be induced by gastric lavage of formula milk powder, cold stress, hypoxia in vivo, or LPS stimulation in vitro [27]. Although the long-term outcome of most premature infants is gradually improving, NEC remains a thorny clinical problem. Therefore, novel treatment strategies are urgently needed. In the present study, IEC-6 cells were stimulated by LPS to establish an intestinal epithelial cell model of NEC in vitro, and the effects of different concentrations of *β*-carotene on NEC cell damage were observed. The cell viability of LPS-treated IEC-6 cells was remarkably decreased compared with the normal cultured cells. However, the cell viability of LPS-treated cells was significantly restored following *β*-carotene (10–30 *μ*M) treatment, indicating that low concentration *β*-carotene has a protective effect on NEC intestinal epithelial cell injury.

NEC is characterized by extensive necrosis and apoptosis of the intestinal epithelium [8]. Apoptosis is a physiological model different from the death of necrotic cells, which plays a significant role in the physiological and pathological processes of many diseases [28]. Experiments show that the change of intestinal epithelial cell apoptosis makes the intestinal tract vulnerable to NEC [29]. Bcl-2 and Bax are two key factors involved in the control of apoptosis through the mitochondrial pathway [30]. In addition, caspase-3 is considered the key protease for coordinating apoptosis [31]. Experimental NEC in rat models showed that the expression of antiapoptotic Bcl-2 decreased while the level of proapoptotic Bax increased [32]. Our results showed that *β*-carotene markedly inhibited apoptosis in LPS-treated IEC-6 cells. Furthermore, we examined the effects of *β*-carotene on the protein expression of cleaved caspase-3, Bax, and Bcl-2. The expression of Bax and cleaved caspase-3 was found to be increased, and the expression of Bcl-2 decreased in the LPS group. However, these proapoptotic effects were notably reversed by *β*-carotene. Taken together, our findings indicate that *β*-carotene may protect IEC-6 cells against LPS by suppressing apoptosis.

Similar to cell apoptosis, autophagy is another process of programmed cell death. It is well known that autophagy is a widespread and highly conservative process of cell degradation, which maintains cell balance by degrading damaged organelles. However, excessive autophagy can lead to cell death [33]. Autophagy plays an important role in the pathogenesis of immunity, infection, inflammation, tumors, angiocardiopathy, and neurodegenerative diseases. LC3 is a key indicator of autophagy. In the process of autophagy, LC3I in cytoplasmic form is transformed into LC3II in phosphatidylethanolamine bound form to promote the formation of autophagy [34]. Therefore, the ratio of LC3II/I is positively correlated with the number of autophagy bubbles, which reflects the autophagy activation of cells to a certain extent [35]. p62, as a marker of autophagy flux, is reduced in the last phase of autophagy, which is negatively correlated with autophagy activity level [36]. In the rat NEC model, autophagy in epithelial cells can be observed by significant activation of autophagy [37]. Importantly, Yang et al. reported that *β*-carotene alleviates LPS-induced intestinal inflammation in rats, which may be related to autophagy [38]. Our findings indicated that *β*-carotene inhibited the autophagy of IEC-6 cells by downregulating LC3II/I levels and upregulating p62 levels. In addition, compared with the LPS group, the number of green autophagy puncta after *β*-carotene treatment decreased. However, these changes induced by *β*-carotene were partially reversed by rapamycin. These results suggest that *β*-carotene can exert protective effects against LPS-induced autophagy in IEC-6 cells.

The PI3K/AKT/mTOR signaling pathway, as an important intracellular medium, is essential in regulating cell growth, metabolism, survival, apoptosis, and autophagy [39, 40]. Moreover, the PI3K/AKT/mTOR signaling pathway is critical to the intestinal survival of NEC [41]. IGF-1 improved the survival rate of newborn rats during NEC by activating the PI3K/AKT/mTOR pathway. On the contrary, targeted silencing of AKT1 markedly increased the mortality of young mice induced by NEC [40]. *β*-Carotene can significantly inhibit advanced glycation end product-induced cell apoptosis and autophagy in H9c2 cardiomyocytes by activating the PI3K/AKT/mTOR signaling pathway [42]. To clarify the regulatory mechanism of *β*-carotene regulated IEC-6 cell apoptosis and autophagy, we used the PI3K inhibitor voxtalisib. It was obvious that voxtalisib reversed the function of *β*-carotene in attenuating apoptosis and autophagy in IEC-6 cells. Subsequently, we studied the changes of key proteins related to the PI3K/AKT/mTOR pathway in IEC-6 cells after *β*-carotene treatment. The results showed that the PI3K/AKT/mTOR pathway was activated, and the expression levels of p-PI3K, p-AKT, and p-mTOR were remarkably increased. However, following voxtalisib cotreatment with *β*-carotene, the expression of p-PI3K, p-AKT, and p-mTOR was distinctly decreased in IEC-6 cells, compared with *β*-carotene treatment alone. Our findings suggest that *β*-carotene was involved in the regulation of LPS-induced apoptosis and autophagy by the PI3K/AKT/mTOR pathway.

## 5. Conclusion

In summary, our findings suggest that *β*-carotene at a low concentration can effectively alleviate NEC intestinal epithelial injury, inhibit apoptosis and autophagy, and protect the NEC intestinal epithelial cell model, but its action pathway is the PI3K/AKT/mTOR pathway. Therefore, *β*-carotene may be a promising molecular-targeted drug for NEC treatment.

## Data Availability

The data used to support the findings of this study are included within the article.

## Abbreviations

NEC: Necrotizing enterocolitis
*β*C: *β*-Carotene
LPS: Lipopolysaccharide
RAPA: Rapamycin
Vox: Voxtalisib.
